# Ultrasonic Imaging and Sensors

**DOI:** 10.3390/s22207911

**Published:** 2022-10-18

**Authors:** Jorge Camacho, Linas Svilainis, Tomás Gómez Álvarez-Arenas

**Affiliations:** 1 Instituto de Tecnologías Físicas y de la Información (ITEFI), Spanish National Research Council (CSIC), 28006 Madrid, Spain; 2 Depertment of Electronics Engineering, Kaunas University of Technology, 44249 Kaunas, Lithuania

## 1. Introduction

Ultrasound imaging is a wide research field, covering areas from wave propagation physics, sensors and front-end electronics to image reconstruction algorithms and software. All these topics are highly related, and it is very common that a novelty in one of them leads to advances in others. It is, indeed, a multidisciplinary field, which gives the opportunity for sharing knowledge and experience between different scientific and technical disciplines, such as physics, materials science, electronics, programming, etc.

A typical ultrasound imaging system is composed of a set of sensors which are excited to generate an acoustic wave in the medium of interest and record the interaction of that wave with matter. This interaction can be based on different physical principles such as scattering, dispersion, absorption, radiation force, etc., and different propagation modes such as bulk, surface or Lamb waves, giving rise to very different types of sensors, imaging modes and techniques. A second key component of an ultrasound system is the front-end electronics, which should be able to independently excite the transducers (usually a large number of them), as well as condition and digitalize the signals received from the medium. Once in the digital domain, the electronic front end is responsible for implementing the beamforming process, which is needed to obtain an image from the raw input data. The requirements for this stage are strongly dependent on the type and configuration of the sensors, the inspected medium’s characteristics, the imaging method used and the real-time demands of the application. Finally, high-level algorithms are usually implemented to display and analyze the obtained images, a topic in which deep learning methods have acquired great relevance in recent times.

This Special Issue entitled Ultrasonic Imaging and Sensors compiles 13 high-quality papers covering most of the research topics in this field. New sensors are proposed, both for direct contact and airborne applications and very different fields, such as medical imaging, concrete structures, molding manufacturing, wind turbines and agriculture, as are new means for testing their integrity. Front-end analog electronics for efficient sensor excitation is also addressed, as well as the optimized design of digital processing architectures for 2D arrays. Furthermore, new imaging methods are presented for improving image quality and inspection velocity, as well as new approaches for image generation and analysis based on deep learning techniques. A classification and short review of the papers in this Special Issue follow.

### 1.1. Sensor Design and Characterization

Two-dimensional (matrix) arrays allow us to generate three-dimensional images without moving the arrays, thanks to their capacity to control the generated wave-field and combine the received signals to and from any point in the space. Their practical limitation lies in the fact that the number of required elements to correctly sample the active aperture (less than half-lambda distance between elements) is much larger than with linear arrays and can easily overpass the current manufacturing capacity of matrix sensors and front-end electronics. One common approach to reduce the system’s complexity, while maintaining the lateral resolution, is to sub-sample the aperture with a reduced number of elements. The consequence of not meeting the half-lambda criterion is an increase in the sidelobes’ level of radiation, which limits the dynamic range of the image. One way of handling this issue is to break the periodicity of the distribution by randomly locating the elements within the aperture, which usually requires implementing computationally intensive optimization algorithms. In [1], Martinez-Graullera et al. propose a new fitting function for this optimization process based on a reduced set of parameters obtained from the spatial distribution of the weights of the co-array representation of the sensor. The key idea behind this work is to avoid calculating the acoustic field for all the candidate arrays during the optimization process, which is computationally expensive. Instead, they obtain three statistic values from the co-array’s representation (occupied surface, standard deviation and kurtosis) which, as they demonstrate in their paper, are highly correlated with the beam pattern of the array and can be obtained with much less computational effort (58 times faster than field calculation for the current implementation). Finally, they analyze the behavior of their method with three examples of ultra-sparse, very sparse and sparse binned arrays, showing a better performance in terms of convergence and final array characteristics than a simple brute-force search method.

The air-coupled ultrasonic probes require a special design approach due to the significant mismatch between piezomaterials and the air. A stack of layers is used in impedance matching. The design and fabrication of these layers require testing at intermediate steps. Outer layers are usually soft, with a number of matching layers being large; therefore, improper handling or exploitation can damage the layers or cause debonding, reducing the probe’s performance. High-excitation voltages are commonly used in air-coupled ultrasound. High stresses can cause electrode detachment or chipping of the piezomaterial. Beam homogeneity is also important, as deviation can lead to incorrect inspection or diagnostics results. Therefore, even the finished transducer requires an evaluation of integrity. Conventional probe inspection procedures such as sensitivity checks, impedance measurements or directivity testing give only integral results; local defects are not displayed nor accounted for. Svilainis et al. [2] proposed a sensitivity map of the transducer element measurement as a new inspection procedure. A sensitivity map is measured using a focused transducer. A focused beam is scanned over the tested probe’s surface and the voltage registered serves as the sensitivity metric. The far-range sidelobes are essential in this application, because the area of the tested probe is higher than the area of the mainlobe of the focused beam. For the transducers, this ratio was almost 500, which produced a 53 dB gain for the sidelobes. The result was that even the low-level, uncorrelated in-phase sidelobes masked the sensitivity map details so the image contrast was reduced and small defects were masked. Another novelty proposed was to use an aperture to mask the sidelobes. The mask was realized as a simple, low-cost hood mounted on the probing transducer. Tight beam focusing revealed small local defects.

Phased arrays are widely used in medical imaging and non-destructive testing in the aeronautical and energy industries. In these cases, relatively high-frequency arrays used for attenuation in the material do not present a significant challenge and high spatial resolutions are demanded. However, the growing demand for phased arrays for more complex materials, such as wood, building materials and concrete, increases the requirement for lower-frequency arrays usable for large-size structures made of materials with large attenuation coefficients, large-sized microstructures or coarse aggregates and, hence, strong scattering. In these cases, arrays in the tens of kHz are needed. In [3], by Ohara et al., the design and method of fabrication of a 32-element phased array with a center frequency of 320 kHz are presented. To improve the transducer’s sensitivity, no backing block and no filler was used. To reduce the crosstalk between piezoelectric elements and dampen the vibration of each element and the concomitant temporal resolution reduction (which become significant problems for backing loss low-frequency arrays), the authors adopted a soft lead zirconate titanate (soft PZT) with a low mechanical quality factor. Moreover, the presented design also includes a combination of thickness and lateral vibration modes in the piezoelectric elements. The performance of the array is tested on a block of concrete with a delamination simulated by inserting a styrofoam plate and on a concrete block with a slit (1 mm width).

Kariminejad et al. [4] reviews the use of ultrasonic techniques for monitoring the process of injection molding, looking at interactions between the polymer and the ultrasonic wave and the use of high-temperature transducers. While most commercial techniques used to monitor these processes rely on point measurements of pressure and temperature, ultrasound techniques represent non-invasive and non-destructive methods, with the potential to provide a larger amount of information related to the mold, the cavity and the polymer melt, and morphology, which affect critical quality parameters in injection molding processes. In [4], the relationship between polymer properties and the propagation of ultrasonic waves is described and the application of ultrasound measurements in injection molding is evaluated. With the presence of high temperatures being a key factor in this application, the principles and operation of both conventional and high-temperature ultrasound transducers (HTUTs), with special emphasis on sol–gel ultrasonic sensors are reviewed together with their impact on the efficiency of the injection molding process.

### 1.2. Front-End Electronics and System Optimization

The signal-to-noise ratio (SNR) in ultrasonic imaging can be improved by operating the transducer at a higher voltage. Transducers used in imaging are usually capacitive. This would result in increased losses in excitation electronics. The optimization of excitation electronics is extremely important in portable and multichannel applications. Both footprint and energy consumption are important when the tight packaging of the transmission electronics is required. Ramos et al. [5] proposed tuned HV capacitive-discharge drivers for multichannel excitation systems. The proposed topology allows the generation of excitation up to 700 V. Selective damping reduces ringing caused by the impedance matching circuit, further improving the bandwidth of the transducers used. A frequency range up to 30 MHz can be obtained with the proposed design. Tight packaging (the demonstrator system has 32 channels) and an optimized electrical performance were achieved by complete system modeling, where even non-linear effects were considered. A net dynamic ranges available (NDRA) value of up to 70–140 dB was achieved (the NDRA of the other currently used systems is 20–70 dB, as estimated by the authors).

The demand for real-time ultrasonic imaging systems has led to array-based systems to using standard equipment in NDT. The Synthetic Aperture Focusing Technique (SAFT) offers reduced hardware requirements at the expense of the frame rate. Both data acquisition and beamforming stages of SAFT imaging require optimization. The acquisition using the Full Matrix Capture (FMC) and processing using Total Focusing Method (TFM) are considered the gold standard to ensure a high-quality image. Nevertheless, hardware parallelism and frame rate are not exploited in full here and the computation and acquisition times increase. Acquisition can be simplified using sparse matrix arrays or sparse non-grid arrays. Sparse non-grid arrays, through which a better optimization of elements can be achieved, are gaining popularity, but the related redundancy identification is a complex issue. A solution was proposed by de Souza et al. [6], who proposed using the Radon transform and the Projection-Slice theorem to identify the redundancy of such arrays. Then, arrays can be designed by using objective parameters; spatial redundancy information helps to reduce the electronic resources and computational resources required for SAFT imaging. The authors demonstrated that this reduction can be achieved without a significant degradation of the original dynamic range. A system designer is given the tool where restrictions on the number of parallel channels and the number of emission elements can be imposed. Additionally, specific elements (if malfunction is detected) can be eliminated, resulting in a fault-tolerant solution. More, such a technique can be used as a metric of the aperture quality. It was shown that, while a smoother co-array shape improves the lateral resolution, the sidelobe level is increased. The solution ACQ(64:16) was proposed, which only uses 16 receiver channels, 64 emitters, and one transmitter, so the further miniaturization of ultrasonic imaging systems for NDT can be achieved.

### 1.3. Imaging Methods for NDT

One of the most relevant parameters for ultrasound imaging in industry is the scanning speed, particularly when inspecting large components with a high spatial resolution and/or for high-volume production lines. As the imaging time is ultimately limited by the time of flight of the ultrasound wave from the transducer to the farthest zone in the component and back to the sensor, a method to increase the image rate is to reduce the number of wave emissions. In a conventional phased-array system, the number of emissions is given by the number of beams needed to correctly sample the inspected component, which, additionally, mainly depends on the array frequency, the active aperture size, and the region of interest extension. In other high-resolution techniques such as the Total Focusing Method (TFM), the number of emissions is equal to the number of array elements, independently of the image characteristics. Depending on the scenario, this figure can be larger or smaller than that created using the phased-array technique, but, in general terms, their magnitudes are similar. A much more efficient method in terms of the number of emissions is plane-wave imaging, which is based on insonifying the medium with a reduced set of plane-waves at different propagation angles. As well as in TFM, a wide emission allows one to generate a complete but low-quality image after each single shot, but the higher energy of the plane-wave with regard to the omni-directional emission of the TFM means fewer low-resolution images are needed to obtain a high-resolution one. As a rule of thumb, plane-wave imaging requires between 5 and 10 times fewer emissions than the phased-array method and TFM to obtain an image with an equivalent quality. In NDT, this translates into 5 to 10 times increase in the scanning speed, which can have a great impact in some applications. Nevertheless, the usual presence of two propagation mediums (coupling and component) and their interface geometry variations during the scan, complicates the calculation of the focal laws needed to generate a plane-wave into the component. In [7], Cosarinsky et al. analyze the problem of generating a plane-wave into an arbitrarily shaped component, giving a closed formulation for the emission delays and for the compensation of the amplitude losses due to the non-homogeneous distribution of the pressure along the wave-front. The authors demonstrate, through simulation and experimentally, that the used approach, which considers the amplitude of the plane-wave constant along its extension, can generate severe distortions in the image. It can modify the relative amplitude of echoes generated by equivalent reflectors and, in extreme cases, it can generate “*blind*” zones, where inspection is not reliable at all. To address this issue, a simple method is proposed to estimate the pressure distribution along the plane-wave, to be used as a compensation factor during image formation or to determine blind zones before inspection.

One of the emerging problems in the field of industrial NDT is the inspection of wind turbine blades. The increasing demand for renewable sources of energy and the increasing number of wind turbines have led to a focus on the maintenance of these structures. The fact that wind turbine blades are subjected to extreme environmental conditions and that they are the most defect-prone and sensitive elements of wind turbines increases the demand for inspection techniques able to detect damage at an early stage, before a problem in a blade can lead to the failure of the whole turbine. In [8], the authors propose a new method for defect detection in wind turbine blades based on the variation of the phase velocity of ultrasonic-guided waves. The main problem in the inspection of wind turbine blades is that the dimension and complexity of these components and the limitation in the applicability and accuracy of some methods make them unsuitable for on-site inspection. The dispersion curves of Lamb waves in turbine blades were simulated, as was the propagation of the selected modes in plates with and without defects. The proposed algorithm was first tested with these simulations. An experimental verification was performed by using a glass fiber-reinforced polymer, a macro-fiber composite transducer operating at 43 kHz to generate the Lamb waves, and a contact-type wide-band piezoelectric transducer. The defect size was 81 mm and the technique demonstrated its capability to properly locate and size the defect.

In addition to the conventional fields of NDT, mainly related to the aeronautical, energy and building industries, other novel fields and applications are continuously appearing. These new fields are extremely interesting as they open up completely new possibilities and applications with significant scaling-up potential. In [9], Parr et al. present an ultrasonic imaging technique for grape yield estimation. Grape yield estimation basically consists of counting grapes and/or bunches of grapes. This is normally performed using manual techniques that are labor-intensive and inaccurate. The use of computer vision techniques is limited by the fact that grapes can be occluded by leaves, branches, and other bunches of grapes. This can be solved by using synthetic aperture radar, but this is an expensive solution. The paper of Parr et al. [9] investigates the potential of using ultrasound to image through leaves and identify occluded grapes. A highly directional low-frequency (40 kHz) ultrasonic array composed of ultrasonic air-coupled transducers and microphones is used to image grapes through leaves. A fan is used to help differentiate between ultrasonic reflections from grapes and leaves. An improved resolution and better detail are achieved with chirp excitation waveforms and the near-field focusing of the array. The results show that it is possible for a low-frequency ultrasound (40 kHz) to penetrate through leaves and generate echoes from the grapes behind, collecting information that can be used for grape cluster detection. In addition, this work also shows that the echoes from grapes and leaves can be distinguished by agitating the leaves using a fan and using the variance of multiple recordings as a filter, and this can be used to reduce the effect of the presence of leaves or occluded bunches of grapes.

### 1.4. Imaging Methods for Biomedical Applications

Plane-wave imaging was originally conceived for medical images, where the propagation velocity can be considered as constant and no refraction problems appear, unlike for NDT. Nevertheless, several authors have proposed variations of the original method for improving image quality as well as reducing system complexity. In [10], Yan et al. propose a novel beamforming strategy based on cascading adaptive weighting and non-linear compounding stages. In the first stage, a minimum variance beamformer was implemented (following the partial generalized sidelobe canceller approach), which contributes to the obtention of a high-resolution image. The second stage, based on a non-linear delay, multiply and sum algorithm, is beneficial for noise suppression and contrast enhancement. Both stages are implemented in low-complexity forms, enabling the implementation of the proposed method in real time, while this is not addressed in the present paper. The performance of the proposed method was evaluated using simulations, phantoms and in vivo images, showing improved image quality and noise immunity compared to simpler beamforming methods such as delay and sum ones, or than each stage algorithm when applied alone instead of in the proposed pipeline.

Ultrasound images can also be used for tissue elasticity evaluation using a shear-wave ultrasound elastography (SWE). The shear waves produced by a focused ultrasound beam are used for tissue elasticity measurement. Mikolajowski et al. [11] concentrated on discussing whether results on lateral abdominal muscles’ (LAMs) thickness and elasticity obtained by an experienced operator, by a non-experienced operator, and with probe compression control produce are different. The problem lies in LAM’s topography: the abdominal area, the region used to assess LAMs, is curved, so it is more challenging to apply the probe without resulting in compression. The conclusion was drawn that differences in measurements are small when the force applied is carefully controlled by the examiner. However, the authors still advise using a probe holder equipped with a pressure sensor when maximum precision is required.

Yet, another novel modality in medical imaging is ultrasound tomography. This is based on emitting and receiving ultrasound signals between sensors located at opposite sides of the object of interest, in a through-transmission configuration. This allows one to reconstruct images of the speed-of-sound and attenuation distribution within the tissue, which provides complementary information to conventional techniques such as B-mode or Doppler. One of the most promising applications of ultrasound tomography is breast imaging for cancer screening. As the speed of sound (SoS) of a tissue is related to its stiffness, which also correlates with tumor malignancy, ultrasound tomography SoS images can be used to complement B-mode images in cancer diagnosis. Furthermore, the radiological dense breast phenomenon, which prevents mammography evaluation in 1 of every 10 women with higher prevalence in younger ones, is not present in ultrasound imaging. This fact could push ultrasound tomography as a complementary technique to mammography in cancer-screening programs. The usual architecture of a breast ultrasound tomography scanner is a ring of independent transducers (typically N ≈ 2.000 elements) that surrounds the breast in a water bath. By sequentially activating pairs of transducers for emission and reception, a dataset of up to N^2^ signals can be acquired, which contains information of a relatively thin slice of tissue in the ring plane. By moving the ring array along the axis of the breast, a 3D dataset of the whole organ can be obtained. The main challenge in these systems is to reconstruct a speed-of-sound or attenuation map of the tissue from the raw data registered by the sensors. Computationally intensive iterative methods are needed, with one of the most promising being the so-called Full Wave Inversion (FWI). Different from simpler approaches that only use the time of flight and peak amplitude, the FWI method uses the received signal’s complete waveform during the optimization process. Being much more demanding that other methods in terms of computation time and convergence, its complexity is directly related to the signal frequency, which determine the spatio-temporal resolution needed during the inversion process. In [12], Robins et al. proposed a novel approach for improving FWI reconstruction performance by training a two-dimensional convolutional neural network able to artificially generate the low-frequency content missing in ultrasound tomography datasets, due to the higher frequency band of the transducers. In particular, this approach helps to avoid the cycle-skipping phenomenon that usually prevents the method converging to a valid solution, and appears when the raw signals lack low-frequency information (below 1 MHz). The CNN was trained with synthetic data obtained from numeric breast models but using a real signal from the acquisition system (3.2 MHz center frequency cardiac phased-array probe) to model the impulse response of the transducer. This way, the resulting CNN is representative of the experimental system used for validation. A multi-modal breast phantom was used to validate this technique. On the one hand, it was demonstrated that the low-frequency extrapolation using the trained CNN significantly improved the quality of the SoS image, avoiding the cycle-skipping problems of the original dataset. Furthermore, the location, size and velocity of the lesions agrees with those obtained by the reference reflectivity images and CT scan.

Other emerging applications of CNNs in medical ultrasound are automatic segmentation and feature extraction. In particular, the presence of clutter and texture can make the manual identification and delimitation of structures in ultrasound images difficult, a task that can could be assisted by automatic algorithms. In [13], Jimenez-Castano et. al. apply these principles to the problem of localizing the nerve structure during peripheral nerve-blocking procedures. This technique is based on the administration of an anesthetic substance around the nerve structure to block, and precise identification is vital for avoiding adverse effects such as the contamination of the blood flow or neurological damage. As attenuation and speckle noise make the visual identification of these structures difficult, automatic segmentation algorithms could help to improve the results, giving physicians a real-time and easy-to-use tool for guiding the procedure. In this paper, the authors propose including a kernel mapping-based layer in the network architecture for improving the generalization capabilities of three deep learning approaches for semantic segmentation: fully convolutional network (FCN), U-Net and Residual Network and U-Net (ResUnet). The kernel mapping layer is based on the random Fourier features extraction method (RFF mapping), and the class activation mapping Grad-CAM++ for providing an efficient data interpretability strategy. The proposed processing pipeline was applied with two ultrasound images’ datasets containing different nerve structures. The results demonstrate that the proposed approach improved the discrimination between the nerve structure and the background in terms of performance parameters such as sensitivity, specificity, intersection over union, area under the ROC curve and geometric mean. Furthermore, RFF-based mapping favors the explanatory capacity of the algorithm, finding relevant maps that highlight image regions related to the nerve structure.
